# A Randomized Trial of Chinese Diaoshi Jifa on Treatment of Dizziness in Meniere's Disease

**DOI:** 10.1155/2014/521475

**Published:** 2014-05-18

**Authors:** Yong-Xin Sun, Yuan Wang, Xunming Ji, Xiaoguang Wu, Yong Zhao, Yuchuan Ding, Mohammed Hussain, Fei Yu, Wenbo Zhao, Jianping Jia

**Affiliations:** ^1^Department of Neurology, Xuan Wu Hospital of Capital Medical University, Beijing 100053, China; ^2^Department of Neurosurgery, Xuan Wu Hospital of Capital Medical University, Beijing 100053, China; ^3^Evidence-Based Medicine Center, Xuan Wu Hospital of Capital Medical University, Beijing 100053, China; ^4^Department of Neurology, Wangjing Hospital, China Academy of Chinese Medical Sciences, Beijing 100102, China; ^5^Department of Neurological Surgery, Wayne State University School of Medicine, Detroit, MI 48201, USA

## Abstract

*Background*. Meniere's disease is characterized by refractory dizziness and hearing disturbance. We aimed to investigate the efficacy and tolerance of Diaoshi Jifa, a Chinese hand skill for treating dizziness in Meniere's disease. *Methods*. An open-labeled, randomized, controlled intervention trial was conducted. Twenty-seven patients diagnosed with Meniere's disease were randomly allocated to control group or experimental group. Both groups were assessed by DHI (dizziness handicap inventory (DHI)) questionnaire score before and within 24 hours of receiving treatment, respectively. *Results*. Twenty-six participants completed the study, and no adverse event was reported due to Diaoshi Jifa treatment. The difference in the DHI scores between baseline and posttreatment reached significant difference in both groups (63.88 ± 19.94 versus 10.25 ± 9.77 and 54.36 ± 17.97 versus 49.6 ± 20.50). Significant difference in DHI scores was observed between the two groups after treatment (10.25 ± 9.77 versus 49.6 ± 20.50). Further investigation of DHI subscales in the experimental group revealed significant improvement posttreatment in the physical domain, functional domain, and emotional domain. Although higher rate of improvement in the emotional domain compared to physical or functional domains was found, the difference was not statistically significant. *Conclusions*. Diaoshi Jifa might be a fast, effective, and well-tolerated method for alleviating dizziness in Meniere's disease.

## 1. Introduction

Meniere's disease is one of the major causes of dizziness syndrome of a peripheral vestibular origin. It is characterized by “recurrent, episodic vertigo associated with hearing fluctuation, hearing loss, aural fullness and tinnitus” [1]. Although Meniere's disease has been attributed to increased pressure within the endolymphatic system [2], the pathophysiology is still controversial [3]. As of now there is no effective medication that can completely treat Meniere's disease.

Diaoshi Jifa is a traditional Chinese approach to treat dizziness arising from various causes. Initiated by Dr. Diao, Diaoshi Jifa has been practiced in China for over 50 years with numerous patients reporting significant improvement in dizziness [4]. Diaoshi Jifa has an advantage of easy application, fast action, and good patient compliance. Although well accepted by the Chinese and widely practiced in traditional medicine, Diaoshi Jifa has not been objectively tested yet with well-designed clinical trials.

The aim of this randomized clinical trial was to examine the effectiveness and tolerance of Diaoshi Jifa in alleviating dizziness symptoms associated with Meniere's disease.

## 2. Methods

### 2.1. Study Design

This study was an open-labeled, randomized, and controlled intervention trial conducted at outpatient clinics of neurology and otorhinolaryngology in the Xuan Wu Hospital of Capital Medical University from January to November 2011. The study protocol was approved by the Ethical Review Board of Xuan Wu Hospital. All participants enrolled in the study signed the informed consent.

### 2.2. Study Participants

Subjects complaining of general dizziness, aged between 20 and 70 years, gave consent at the outpatient clinic and were then screened for inclusion criteria (meeting the American Academy of Otolaryngology-Head and Neck Surgery Committee on Hearing and Equilibrium criteria for probable Meniere's disease entailing a “washout period” of at least 5 days of any prior treatment before enrollment) [5] and exclusion criteria (illness of other systems that is not appropriate for manual treatment). A professional team consisting of 4 neurologists and 1 otolaryngologist performed physical examinations and prescribed pure tone audiometry and brain magnetic resonance imaging (MRI) for all subjects for the purpose of establishing a diagnosis or differential diagnosis. We collected the baseline characteristics of the participants, including sex, age, recurrent vertigo, family history, injury history, and smoking history.

### 2.3. Intervention

Participants in the control group received intravenous Ginkgo Injection (Ginkgo 20 mg, Beijing Double-Crane Pharmaceutical Business Co., Ltd., China) of 20 mL once a day and oral betahistine mesylate tablet (Merislon, Eisai Co., Ltd., China) of 6 mg 3 times a day. Participants in the experimental group received Diaoshi Jifa treatment, followed by the medicinal regimen identical to that used in the control group.

Diaoshi Jifa treatment consists of 3 major procedures: finger press of the acupuncture points, massage of the acupuncture points, and dynamic manipulation of the acupuncture points.

**Figure 1:**
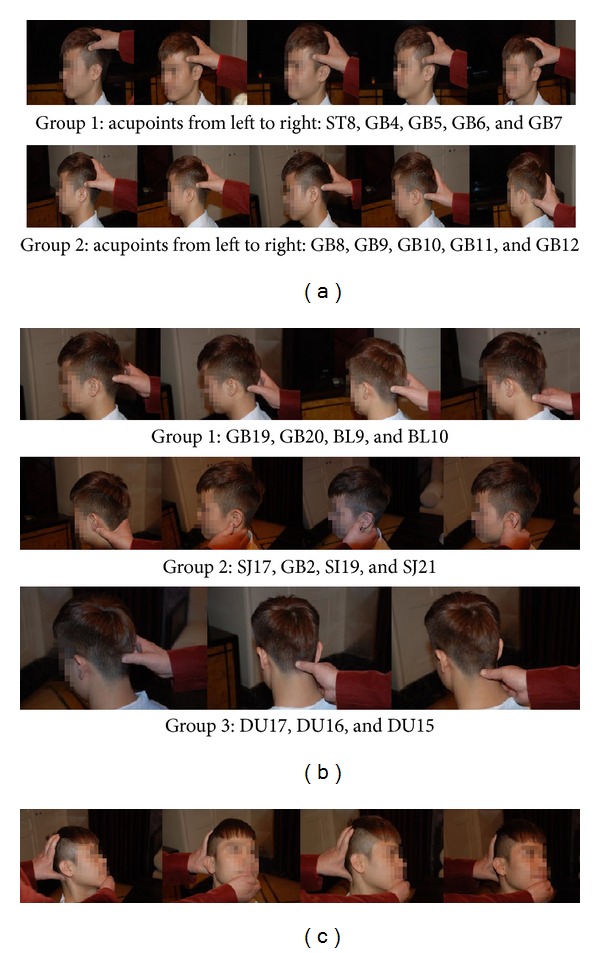
Diaoshi Jifa treatment procedure (the patient in the pictures signed the consent form). (a) A one-time finger press of the provided acupoints in sequence and repetition of the aforementioned motion 3 to 5 times. (b) Massaging of the acupoints clockwise for 3 cycles involving the provided sequence and repeating the sequence 3−5 times. (c) Dynamic manipulation of the acupuncture points in a two-step manner. Use thumb of one hand to press the acupoints “Wan Gu” GB12 (a) and “Tian Zhu” BL10 (b) and support the head with the other 4 fingers. Use the other hand to hold the chin 15° upward. Slightly rotate the head with both hands 2 to 3 times. One should feel the thumb move in the acupoint zones.

*Step  1.* A one-time finger press of the following acupoints in sequence and repetition of the aforementioned motion 3 to 5 times. (1) Press the first group of acupoints: from ST8 to GB4, GB5, GB6, and GB7. (2) Press the second group of acupoints: from GB8 to GB9, GB10, GB11, and GB12 (Figure 1(a)).

*Step  2.* Massage the acupoints clockwise for 3 cycles involving the following sequence and repeat the sequence 3−5 times. (1) Massage the first group of acupoints: from GB19 to GB20, BL9, and BL10. (2) Massage the second group of acupoints: from SJ17 to GB2, SI19, and SJ21. (3) Massage the third group of acupoints: from DU17 to DU16 and DU15 (Figure 1(b)).

*Step  3.* Dynamic manipulation of the acupoints in a two-step manner is as follows. (1) Use thumb of one hand to press the “Wan Gu” GB12 and support the head with the other 4 fingers. Use another hand to hold the chin with a tilt of 15° upward. Slightly rotate the head with both hands 2 to 3 times. One should feel the thumb move in the zone of the acupoint. (2) Use the thumb of one hand to press the “Tian Zhu” BL10 and support the head with the other 4 fingers. Use another hand to hold the chin with an upward tilt of 15°. Slightly rotate the head with both hands 2 to 3 times. One should feel the thumb move in the acupoint (Figure 1(c)).

### 2.4. Outcome Measures and Quality Assurance Procedure

Participants were objectively assessed for dizziness by the dizziness handicap inventory (DHI) after randomization into either the experimental or control group [6]. DHI contains 25 items with a total score of 100 points (4 points for each item). Higher scores correlate with more severity of a handicap [7]. The scale is comprised of a mix of questions: 7 physical, 9 functional, and 9 emotional questions. DHI was assessed again within 24 hours of the first day's treatment.

Various measures were implicated for quality assurance (external quality assessment (EQA)) involving the sampling scheme which was used to determine the sample size. The EQA was conducted by experts including statisticians from the Chinese Center for Disease Control and Prevention (CDC), Ethical Review Board Member from Xuan Wu Hospital, and clinicians from the Department of Neurology, Xuan Wu Hospital and Dongzhimen Traditional Chinese Hospital. Sampling for EQA was performed before, during, and at the end of the study, with each assessment meeting the quality standard. The information recorded by the interviewer was checked at the end of the study to ensure completeness. It was completed and met the quality standards.

### 2.5. Statistical Analysis

Statistical analysis was performed using SPSS 17.0 package. Between-group differences in demographic and baseline variables were tested using a one-way analysis of variance and independent-sample *t*-tests. Intervention effects after treatment were compared by means of independent-sample *t*-tests and paired *t*-tests (with 95% confidence intervals (CI)). Independent-sample *t*-tests were used to compare between experimental and control groups, and paired *t*-tests were used to examine within-group changes from baseline to posttreatment. The changes included the means of total DHI scores and three subscale scores. Subscale score changes were compared within the experimental group by means of change rate comparison: (posttreatment score − pretreatment score)/pretreatment score × 100%. A two-sided *P* value of less than 0.05 was considered statistically significant.

## 3. Results

**Figure 2:**
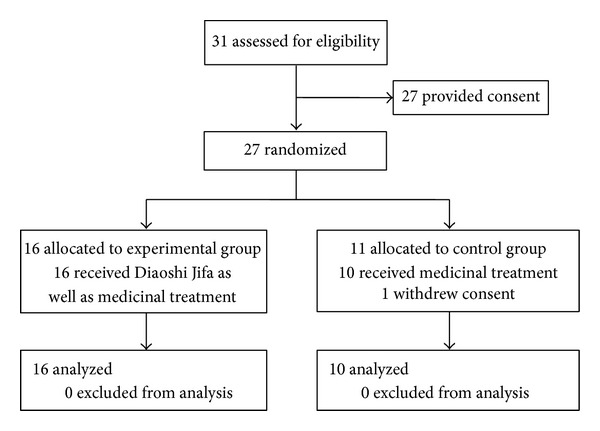
Flow diagram of subjects through the protocol.

Twenty-seven subjects were enrolled and randomized in the study to receive either the medicinal treatment (control group) or Diaoshi Jifa in addition to medicinal treatment (experimental group). One participant in the control group voluntarily terminated his enrollment in the study before treatment started, leaving 26 participants. The flow of participants is illustrated in Figure 2.

### 3.1. Baseline Characteristics of the Participants

Table 1 shows the baseline data of the 27 participants. There were no significant differences seen in the baseline demographic variables between the experimental and control group.

### 3.2. Primary Outcomes

Table 2 shows the DHI evaluation data in the 26 participants before and after treatment application. The baseline DHI score was 63.88 ± 19.94 for the experimental group and 54.36 ± 17.97 for the control group, and there were no significant differences between the two groups (*P* = 0.217). In the experimental group, DHI score changed dramatically from 63.88 ± 19.94 at baseline to 10.25 ± 9.77 after application of both medicinal and Diaoshi Jifa treatments (*P* < 0.001); in the control group, the change in the DHI scores was less dramatic on comparing scores before and after medicinal treatment alone (54.36 ± 17.97 versus 49.6 ± 20.50, *P* = 0.029). On comparison of the DHI scores posttreatment, scores differed significantly between the two groups (49.6 ± 20.50 for the control group versus 10.25 ± 9.77 for the Diaoshi Jifa group). We further evaluated substratified scale scores in both groups, as shown in Table 3. Participants in the Diaoshi Jifa group showed significant improvement after treatment in physical domain (2.25 ± 2.91 versus 19.13 ± 8.67, *P* < 0.001), functional domain (6.50 ± 5.90 versus 25.63 ± 8.17, *P* < 0.001), and emotional domain (1.50 ± 3.46 versus 19.13 ± 8.70, *P* < 0.001). In contrast, participants in the control group showed no significant changes in DHI subscale scores in either physical domain (*P* = 0.122), functional domain (*P* = 0.068), or emotional domain (*P* = 0.126). Between-group analysis showed no significant difference between Diaoshi Jifa group and control group at baseline in any of the 3 subscales (*P* = 0.511 for physical domain, *P* = 0.411 for functional domain, and *P* = 0.479 for emotional domain, resp.). In contrast, posttreatment comparison of the subscale scores between the experimental and control group showed a significant difference in physical domain (*P* = 0.001), functional domain (*P* < 0.001), and emotional domain (*P* = 0.001), respectively.

**Figure 3:**
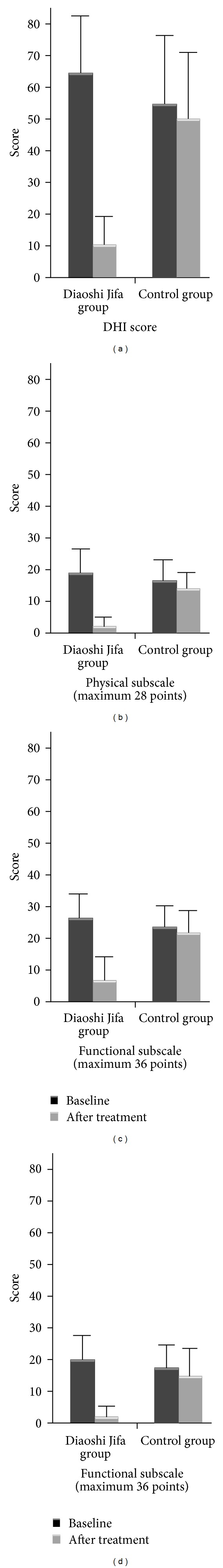
The DHI scores and the three subscale scores. In the Diaoshi Jifa group, the change rate was 88.3% for physical subscale, 74.7% for functional subscale, and 92.2% for emotional subscale.

An additional analysis was conducted to determine whether participants within the Diaoshi Jifa group had similar improvement in the 3 subscales of DHI after treatment. As shown in Figure 3, the absolute value of DHI score change rate was 88.3% for physical domain, 74.7% for functional domain, and 92.2% for emotional domain. Although the emotional domain was most affected by the treatment in the Diaoshi Jifa group when compared to the physical and functional domains, the difference was nevertheless statistically insignificant.

## 4. Discussion

This randomized, controlled trial shows that Diaoshi Jifa has a beneficial effect on alleviating dizziness in patients with Meniere's disease. It was seen that participants in control and experimental groups at baseline had similar DHI scores. After one day of treatment, patients in the experimental group showed significant improvement in DHI scoring, whilst a marginal improvement was seen in the control group. There was a significant difference in DHI scores between the experimental group and the control group within 24 hours after the first day's treatment. All the 16 participants in the experimental group completed the entire study and no adverse events were reported, validating the safety and good compliance of Diaoshi Jifa treatment for dizziness in patients with Meniere's disease.

Meniere's disease is a complex syndrome that originates from the inner ear; however, its etiology and pathophysiology remain controversial. Endolymphatic hydrops, endocrine dysfunction, congenital abnormalities, and psychosomatic factors have all been proposed to cause Meniere's disease, but an effective treatment to this condition still eludes [8]. From a long-term perspective Meniere's disease holds devastating consequences when it comes to hearing ability [9]. Each episode is rather self-limited and medicinal treatment such as betahistine and* Ginkgo biloba* has shown to have ambivalent results in alleviating dizziness [10]. Therefore we decided to evaluate the effectiveness of first-time application of Diaoshi Jifa over a short period (24 hours) of time.

Diaoshi Jifa stems from traditional Chinese approach to treat dizziness in patients with chronic disorders of multiple etiologies. Innovated by Dr. Diao, the method has been in practice in China for over 50 years. The principle theory explaining the effectiveness of Diaoshi Jifa stems from its ability to reconstitute a neurovascular response in the cervical and scapular areas based on muscle relaxation and acupuncture point manipulation [4]. Our data validated the effectiveness of Diaoshi Jifa by rapidly relieving dizziness in patients with Meniere's disease.

The self-perceived handicap in Meniere's disease patients was evaluated by 3 subscales of DHI: physical, functional, and emotional domains. In addition to the overall improvement in DHI scores, participants in the Diaoshi Jifa group showed significant improvement in each subscale after receiving treatment (*P* ≤ 0.001 for all subscales). Though not statistically significant, emotional subscale had the greatest score change rate compared to the physical and functional subscales (−92.2% versus −88.3% and −74.7%, *P* > 0.5). In fact, after treatment with Diaoshi Jifa, most participants claimed to have felt a more relaxed feeling, especially in the cervical and scapular muscles, in addition to a clear mind and even better visual acuity. Jacobson and Newman [6], in their article of the development of 25-item DHI, reported that, although the overall internal reliability value (Cronbach's *α* coefficient) was rather high (0.89), the emotional subscale had the lowest Cronbach *α* coefficient (0.72 versus 0.78 for physical subscale and 0.85 for functional subscale). Emotional subscale was also affected significantly by the frequency of dizziness episodes [11]. Compared with the relatively objective questions listed in the physical subscale, subjects tended to respond variably to questions in the emotional subscale such as “Because of your problem, do you feel frustrated?” These results were in accordance with the widely reported psychosomatic property of Meniere's disease [12].

The present study has some limitations. Firstly, the open-labeled property of the clinical trial made those patients who seek traditional Chinese medications more predisposed to enrolling, thus contributing towards a selection bias. Secondly, this study was designed to be conducted in a single clinical center instead of a multicenter clinical trial. The reason is attributed to Dr. Diao, being the innovator of Diaoshi Jifa, ensuring a homogenous manipulation to assess preliminary analysis of treatment effect and thus restricting the study to a single center to overlook a proper execution of the manipulation techniques. Thirdly, our study focused only on the short-term effectiveness of Diaoshi Jifa (within 24 hours after one-time treatment). Though positive results were seen rapidly by Diaoshi Jifa, it should be noted that patients usually get symptomatic alleviation after several days of initiating Meniere's disease medications.

In conclusion, our preliminary data showed that Diaoshi Jifa (a traditional Chinese approach) combined with medical regimen was effective in relieving dizziness symptoms in patients with Meniere's disease. In a short period of time (within 24 hours), patients showed symptomatic relief both functionally and emotionally from the treatment. The advantage of a relatively fast effect in addition to its safety makes Diaoshi Jifa a viable alternative to conventional medical treatment for Meniere's disease. Multicentered trial assessing its effectiveness on a larger scale is warranted.

## Figures and Tables

**Table 1:** Demographic and clinical characteristics of the study participants at baseline.

Variables	Diaoshi Jifa group (*N* = 16)	Control group (*N* = 11)	*P*
Female sex: number (%)	11 (68.8)	5 (45.5)	0.264
Age	50.1 ± 15.6	54.2 ± 8.7	0.356
Recurrent vertigo: number (%)	11 (68.8)	7 (63.6)	1.000
Family history: number (%)	4 (26.7)	2 (18.2)	0.942
Injury history: number (%)	6 (37.5)	2 (18.2)	0.405
Smoking history: number (%)	4 (25.0)	5 (45.5)	0.411

Plus–minus values are means ± SD. Differences in demographic and baseline variables were tested with a one-way analysis of variance and independent-sample *t*-tests. There were no significant between-group differences in any baseline characteristics.

**Table 2:** Changes in dizziness handicap inventory (DHI) score in the participants.

DHI score	Diaoshi Jifa group (*N* = 16)	Control group (*N* = 10)	Between-group difference (95% CI)	*P*
Baseline	63.88 ± 19.94	54.36 ± 17.97	−9.51 (−24.98, 5.96)	0.217
After treatment	10.25 ± 9.77	49.6 ± 20.50	39.35 (24.20, 54.50)	<0.001*
Within-group difference (95% CI)	−53.63 (−42.87, −64.38)	−6.80 (−0.87, −12.73)		
*P*	<0.001**	0.029**		

Plus–minus values are means ± SD. The paired *t*-test was used for within-group comparison, while independent *t*-test was used for between-group difference. *Significantly different from the control group (*P* < 0.01). **Significantly different from the baseline (*P* < 0.05).

**Table 3:** Changes in dizziness handicap inventory (DHI) subscale scores in the participants.

DHI subscale scores	Diaoshi Jifa group (*N* = 16)	Control group (*N* = 10)	Between-group difference (95% CI)	*P*
Physical subscale (maximum 28 points)				
Baseline	19.13 ± 8.67	16.80 ± 8.60	−2.33 (−4.87, 9.52)	0.511
After treatment	2.25 ± 2.91	14.20 ± 8.30	11.95 (5.91, 17.99)	0.001*
Within-group difference (95% CI)	−16.88 (−12.35, −21.40)	−2.6 (−0.84, 6.04)		
*P*	<0.001**	0.122		
Functional subscale (maximum 36 points)				
Baseline	25.63 ± 8.17	23.00 ± 7.07	−2.63 (−3.84, 9.10)	0.411
After treatment	6.50 ± 5.90	21.20 ± 7.67	14.7 (9.19, 20.21)	<0.001*
Within-group difference (95% CI)	−19.13 (−14.41, −23.84)	−1.8 (−0.16, 3.76)		
*P*	<0.001**	0.068		
Emotional subscale (maximum 36 points)				
Baseline	19.13 ± 8.70	16.60 ± 8.75	−2.53 (−4.73, 9.78)	0.479
After treatment	1.50 ± 3.46	14.20 ± 9.97	12.7 (6.15, 19.25)	0.001*
Within-group difference (95% CI)	−17.63 (−13.36, −21.89)	−2.4 (−0.82, 5.62)		
*P*	<0.001**	0.126		

Plus–minus values are means ± SD. The paired *t*-test was used for within-group comparison, while independent *t*-test was used for between-group difference. Subscale score changes were compared within the experimental group by means of change rate comparison. *Significantly different from the control group (*P* < 0.05). **Significantly different from the baseline (*P* < 0.05).
